# An Optoelectronic System for Measuring the Range of Motion in Healthy Volunteers: A Cross-Sectional Study

**DOI:** 10.3390/medicina55090516

**Published:** 2019-08-22

**Authors:** Francesc Medina-Mirapeix, Rodrigo Martín-San Agustín, Germán Cánovas-Ambit, José A. García-Vidal, Mariano Gacto-Sánchez, Pilar Escolar-Reina

**Affiliations:** 1 Department of Physiotherapy, University of Murcia, 30001 Murcia, Spain; 2 Department of Physiotherapy, University of Valencia, 46010 Valencia, Spain; 3 Department of Physical Therapy, EUSES University of Girona, 17190 Girona, Spain

**Keywords:** lower extremity, range of motion, reproducibility of results, articular goniometry

## Abstract

*Background and Objectives*: Within the clinical evaluation of multiple pathologies of the lower limb, the measurement of range of motion (ROM) of its joints is fundamental. To this end, there are various tools, from the goniometer as a reference to more recent devices such as inclinometry-based applications, photo capture applications, or motion capture systems. This study aimed to assess the validity, intra-rater, and inter-rater reliability of the VeloFlex system (VS), which is a new camera-based tool designed for tracking joint trajectories and measuring joint ROM. Materials and Methods: Thirty-five healthy volunteers (16 females; aged 18–61 years) participated in this study. All participants were assessed on two separate occasions, one week apart. During the first assessment session, measurements were obtained using a goniometer and the VS, whereas, in the second session, only the VS was used. In each assessment session, nine active movements were examined. For each movement, three trials were tested, and the mean of these three measures was used for analysis. To evaluate the concurrent validity and agreement, the Pearson correlation coefficient (r) and Bland-Altmann plots were used. Intra-rater and inter-rater reliability were evaluated using intra-class correlation (ICC), standard error of measurement (SEM), and minimal detectable change (MDC). Results: Both devices showed excellent correlations for all movements (r ranged from 0.992 to 0.999). The intra-rater reliability of the VS was excellent (ICC ranged from 0.93 to 0.99), SEMs ranged from 0.53% to 2.61% and the MDC ranged from 0.68° to 3.26°. The inter-rater reliability of the VS was also excellent (ICC ranged from 0.88 to 0.98), SEMs ranged from 0.81% to 4.76% and the MDC ranged from 2.27° to 4.42°. *Conclusions*: The VS is a valid and reliable tool for the measurement of ROM of lower limb joints in healthy subjects.

## 1. Introduction

Measurement of range of motion (ROM) plays an important role in the clinical examination of patients with lower limb disorders, such as hip arthroplasty or acute ankle sprain [1,2]. Universal goniometers (UG) are widely used in clinical practice to measure ROM of lower limb joints [3] and can be considered as a reference standard [4]. However, numerous valid and low-cost technology tools (e.g., inclinometry-based applications or photo capture applications) are also currently available for clinicians [5]. The photo capture software available often requires an examiner to mark landmarks on a photo of a joint at the end of ROM. Based on these data, the software calculates the angle of the drawn lines [6]. This software, which can be downloaded from the Internet for free or a reduced cost, provides highly reliable and valid data [7]. However, one major drawback of these tools is that they require a processing analysis after creating the photo, which can be time-consuming.

Alternatively, ROMs have also been measured with motion capture systems using electromagnetic or optoelectronic devices that are highly accurate in tracking markers [8]. However, these systems have not proved to be suitable in a clinical setting, as traditionally they are expensive with complex configurations and requirements (e.g., they use six or more cameras and have a latency time) [9]. Moreover, many of these systems also require a post-processing analysis after motion capture [8]. Nevertheless, since Nintendo introduced the WiiRemote^TM^ (Nintendo, Kyoto, Japan) into the market, which is capable of tracking an active marker that emits infrared light [10], many of these disadvantages have been reduced, and data can be acquired in real-time [11]. Indeed, this system has been validated in rehabilitation settings [12,13] and for kinematic analysis [11].

In spite of being a handy and easy to use tool, the utility of the WiiRemote^TM^ to track points is limited to certain lighting conditions and a sampling rate of 100 samples per second [14]; thus new optoelectronic systems have emerged [15,16]. The VeloFlex system (VS) (Deportec, Murcia, Spain) is a new tool that can track up to four markers and provides real-time feedback with zero latency time. The core of the VS is a monochrome IRcam like the one used by WiiRemote^TM^ [14,17]. By lacing the axis of the camera lens perpendicular to the plane of motion, the camera provides the position and distance between two markers with a full-scale accuracy of 1% and a repeatability of 0.1%, which allows the VS to determine angles between segments with an accuracy of 0.1°. The aim of the present study was to assess the concurrent validity of the VS and the UG for measuring lower limb ROM on healthy subjects and to examine their intra and inter-rater reliability.

## 2. Materials and Methods

### 2.1. Participants and Study Design

Thirty-five healthy volunteer subjects with no history of pathology, surgery, or permanent impairments affecting the lower limb were recruited from a sports center (Gimnasio Misan, Totana, Murcia, Spain). Participants were users of the center recruited through advertising. All those who initially contacted the researchers to participate in the study were ultimately examined in the same sports center. The exclusion criteria were people with pain or less than 75% of standard ROM in any examined joints, or any inability to perform active lower limb movements. All participants provided informed consent and completed an information sheet prior to data collection, including demographic (age, gender) and anthropometric measures (height, weight). The experimental protocol was approved by the Ethics Committee of the University of Murcia (Spain) (CEI-2263; approved on 06/03/2019). Data collection was performed between March and April of 2019.

All participants were tested on two separate occasions in a room at a similar environmental temperature and at the same time of day. During the first session (S1), all movements were measured by the UG and VS in order to examine concurrent validity. In session two (S2), one week apart, measurements were only performed using the VS, in order to examine intra-rater and inter-rater reliability within the same session. An experienced examiner in the use of both the UG and the VS conducted all ROM movements at S1 and S2 and read the UG values. At S2, ROM was additionally measured by an examiner without previous experience with the VS. This examiner was instructed in the use of the VS during a theoretical and practical training session. This consisted of theoretical information on the Veloflex operating mechanism, what it is, and what it is used for. In addition, the examiner was informed of all the movements that were to be evaluated as well as the reference points that should be considered for each movement. Subsequently, the protocol to be used during the measurement process was explained, together with aspects such as breaks, repetitions, etc. Finally, the practical explanation consisted of the execution of the measurement protocol on a volunteer under the supervision of examiner 1. The total duration of the training session ranged between 35–40 min. The order of measurements performed by these examiners was randomized in S2. An assistant researcher read the VS values during all sessions in order to avoid bias. The examiners and the assistant were unaware of the other examiner’s scores.

### 2.2. Instrumentation

**Figure 1:**
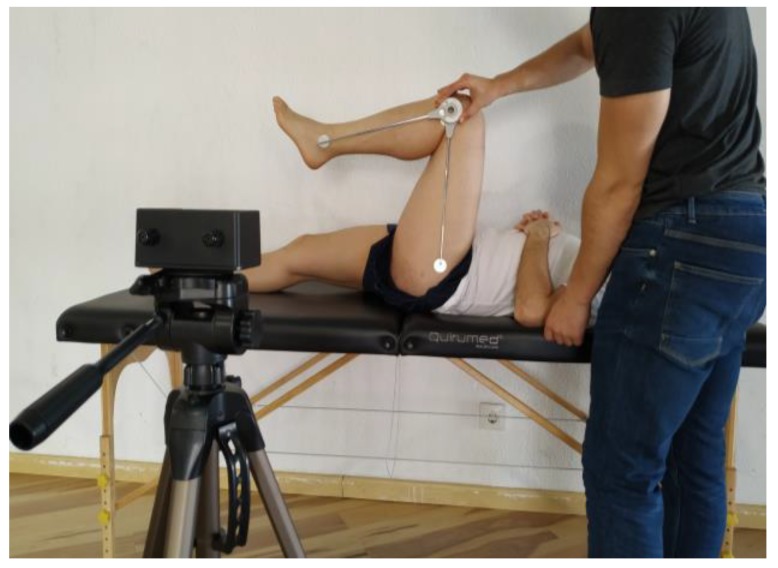
Measurement of knee joint range of motion for flexion.

A simple long-arm UG (Orthopedic Equipment Co., Bourbon, IN, USA) with a 360° scale, marked in one-degree increments, was used. The VS (Deportec, Murcia, Spain) is an optoelectronic system which acquires position data that provides knowledge on the angular amplitude at each moment during the performance of the joint movement, in order to obtain variables, such as the ROM. The VS comprises three elements: An infrared camera with a tripod, a laptop, and markers that are easy to attach to the skin. The camera, by the reflection of the infrared light, obtains the position (Cartesian coordinates (X, Y)) of the geometric center of the reflective marker, thus differing from video recordings done using traditional cameras. The camera was placed on an adjustable tripod stand at a 1–1.5 m distance from the participant and located at the height of the joint that was being examined (Figure 1). The VS can determine the angle formed by two bone segments (with three reference points: One joint axis and two references) or, alternatively, between a bone segment and the horizontal/vertical line drawn on the joint axis. Depending on the camera’s proximity to the joint, the system’s accuracy has been reported to be between 0.1° and 1° [16], without influencing other operating system properties.

### 2.3. Procedures

During each measurement session, nine movements were examined. For each movement, three trials were tested, and the mean of three measurements was used for analysis. The tests were performed in the following order: Hip, knee, and ankle movements. Within each joint, the order of testing was randomized, however, pairs of movements on the same plane (e.g., abduction and adduction, internal then external rotation, etc.) were always measured consecutively. All test positions and references for goniometer alignments were chosen according to the recommendations by Norkin and White (2016) [18]. These references were also used for the VS markers.

The test protocol for preparing participants and performing the VS measurements was strictly identical for the two sessions and examiners. Before testing the movements in each joint and cardinal plane, a physiotherapist trained participants on the protocol requirements (movement directions, pauses, repetitions, and sequences) and stabilizations [18]. Patients completed a warm-up trial by performing five full movements. If the examiner observed any compensatory pattern due to a lack of stabilization, participants were asked to perform an additional movement without compensation.

Initially, for the measurement procedure with the VS, the examiner placed the VS markers on representative anatomical landmarks on the joint axis and both the midline of moveable and stationary segments while the participants maintained the maximal joint range in a given primary direction. For specific tasks, the latter was substituted by using a horizontal or vertical axis at the joint axis. Secondly, after returning to the initial position, the VS was switched on and participants were asked, once again, to move toward the maximal range, maintain this position for three seconds and then return to a neutral position. After a five second pause, this movement pattern was repeated three times. After five seconds, the opposite direction was measured in a similar manner. A 60 s rest period between cardinal planes or joints was provided in order to change the position of the camera, if necessary.

The measurements were performed consecutively with both the VS and the UG in S1, as we also examined the concurrent validity while the participants maintained maximal range for three seconds. The testing order of both instruments was assigned in a random order and retained during the three trials. When the UG was used first, the examiner removed the goniometer, maintaining the maximal joint range, then subsequently placed the markers and performed the VS measurement. In the opposite case, after the VS measurement, the examiner removed the markers, then, retaining the maximal range performed the UG measurement.

### 2.4. Data Analysis

All statistical analyses was performed using SPSS version 24.0. The participant’s demographic information and the ROM data were summarized using means and standard deviations (SDs) or percentages, as appropriate. To evaluate the concurrent validity and agreement between the measurements of the two devices (VS and the UG), the Pearson correlation coefficient (r) and Bland-Altman plots were used, respectively. Moreover, the following statistics were calculated: Upper and lower limits of agreement (LoA), the mean and the SD of the difference between the two devices (these concepts were termed ‘bias’ and ‘imprecision’, respectively), and their respective percentages compared to the UG scores. Relative reliability is the degree to which individuals maintain their position in a sample with repeated measurements. To analyze the relative intra and inter-rater reliability, intraclass correlation coefficients (ICCs) were calculated. Absolute reliability is the degree to which repeated measurements vary for individuals, expressed as the standard error of measurement (SEM), and the minimal detectable change (MDC), with a 95% confidence interval. The SEM and MCD were calculated as:

$$\mathrm{SEM}=\mathrm{SD}\sqrt{1}-\mathrm{ICC}$$

where SD is the SD of all participant measurements.

MDC=SEM×1.96×√2

The sample size was calculated using the formula for reliability studies based on confidence intervals (CIs) described by Streiner and Norman [19]. With the number of instruments (k) equal to 2, the CI around r (the reliability coefficient) of 0.05, and an estimated r of 0.95, the sample size (*n*) was calculated as a minimum of 25 participants. However, ultimately, we included 10 more subjects in the final sample in order to increase the study power.

## 3. Results

Table 1 shows the participants’ characteristics. All participants (16 females) completed all the tests. Their ages ranged from 18 to 61 years, with a mean age of 34.8 years (SD = 12.5). Their mean BMI was 25.3 kg/m^2^ (SD = 5.9).

The results of the ROM extracted in S1 from both the VS and UG instruments are reported in Table 2. The mean lower limb ROM ranged from 20.27° to 139.88° for the goniometer, and 20.42° and 139.97° for the VS, both for ankle dorsiflexion and for knee flexion, respectively.

Both devices showed an excellent correlation for all movements (r’s range from 0.992 to 0.999).

**Figure 2:**
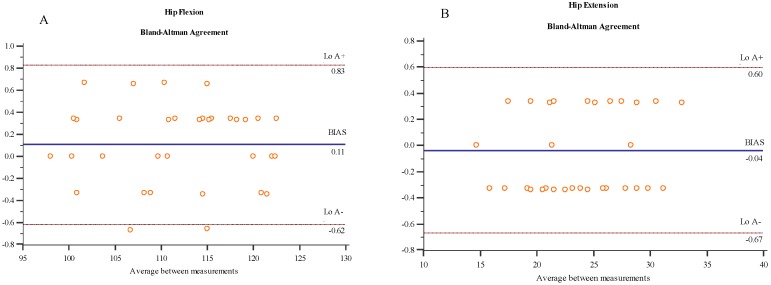

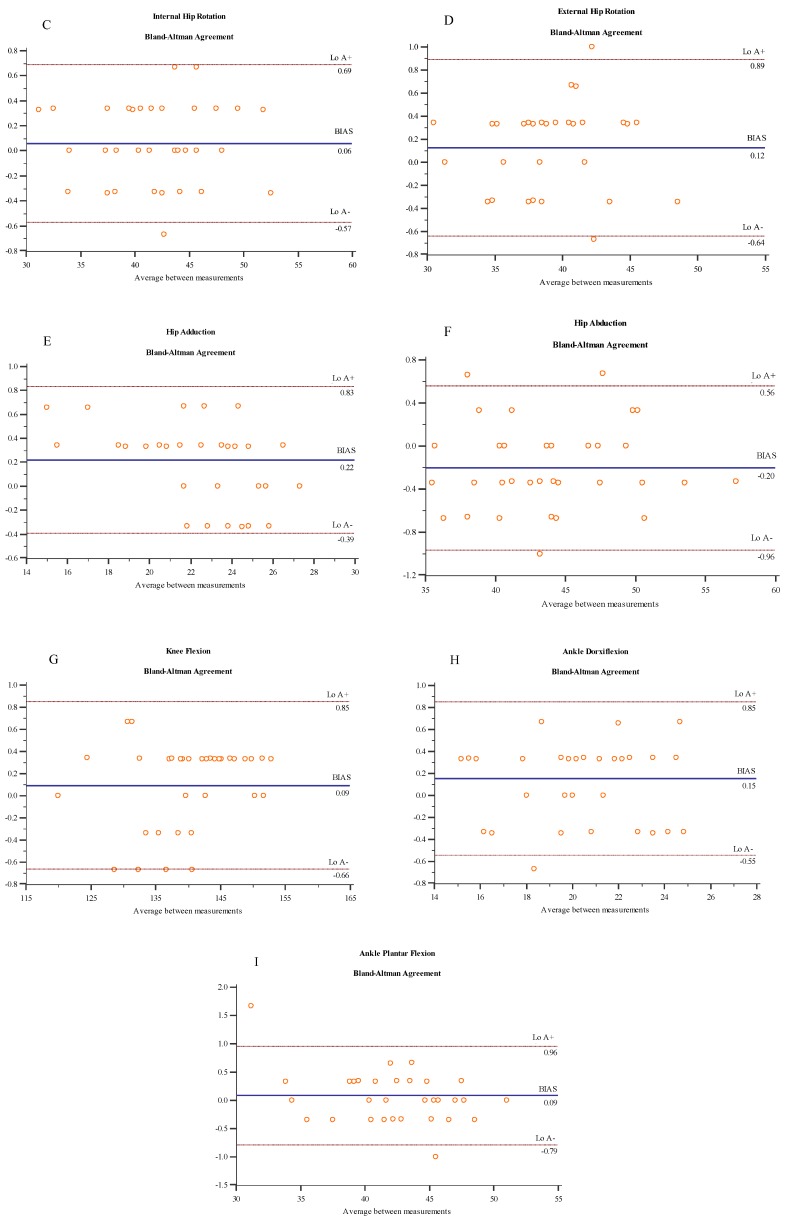
Bland-Altman plots for Veloflex and goniometer measurements during: (**A**) Hip flexion; (**B**) hip extension; (**C**) hip internal rotation; (**D**) hip external rotation; (**E**) hip adduction; (**F**) hip abduction; (**G**) knee flexion; (**H**) ankle dorsiflexion; (**I**) ankle plantarflexion.

The Bland-Altman plots are provided in Figure 2. Both devices provided an excellent agreement of ROMs across movements with LoA+ and LoA− ≤ 1° in all movements compared to measures with the UG, and a small mean ‘bias’ (≤1.1%) and ‘imprecision’ (SD ≤ 1.8%). Knee flexion showed the lowest ‘bias’ (0.1%) and ‘imprecision’ (0.3%) values. In contrast, ankle dorsiflexion was the movement with the highest values (0.8% and 1.8%, respectively).

Table 3 and Table 4 show the ROM values reported by the VS in session two for the two examiners and data from the intra-rater and inter-rater reliability analysis, respectively. The intra-rater reliability of the VS when measuring lower limb ROM was very good, with excellent ICCs (range from 0.93 to 0.99). The SEMs ranged from 0.53% to 2.61%, and the MDC ranged from 0.68° to 3.26°. The inter-rater reliability was also high for all tests, with excellent ICCs (range from 0.88 to 0.98). The SEMs ranged from 0.81% to 4.76%, and MDC ranged from 2.27° to 4.42°.

## 4. Discussion

The present study found that the VS is a valid and reliable alternative method to the UG for the measurement of active ROM in lower limb joints when testing healthy individuals without impairments. Moreover, it showed that basic training in the use of the VS for an inexperienced examiner appears to be sufficient in order to achieve reliable measures.

For all examined ROM, the VS exhibited an excellent correlation and agreement with the UG, with a very low mean bias (1.1%) and imprecision (1.8%). The differences between the correlation coefficient for all tests were minimal (r > 0.992), as well as between the agreement statistics. Nevertheless, the percentage of mean bias was higher for hip adduction than hip abduction, and also for ankle dorsiflexion compared to plantarflexion. These differences between the two movements of the same plane can be explained by the large differences between their ROM. Likewise, previous studies have also obtained better results with a larger ROM compared to a smaller one [20,21]. Overall, our findings were slightly better than those of previous studies which examined the validity of alternative methods to the traditional UG for measuring ROM of one or more lower limb joints in healthy people [4,5,22]. For example, our study showed lower LoAs than several studies that compared the UG and knee joint measurements with either smartphone applications, based on photography captures [5], or internal accelerometers [4,22].

The intra-rater reliability using the VS obtained in this study was excellent (ICC > 0.93) for all movements. The differences of the ICCs and SEMs between movements were minimal (<2.61%), and the percentages of SEMs were especially similar between movements in the same plane (e.g., hip flexion and extension). Thus, from a clinical perspective, the VS is a reliable method when used by the same examiner. Moreover, according to MDCs, limited changes in ROM (e.g., above 3° for hip flexion) can be considered to be “real” changes measured using the VS in healthy individuals. Other instruments, such as the UG [21,23], digital inclinometers [23], and newly validated instruments, such as smartphone applications [4,5] have also shown high ICCs of intra-rater reliability for lower limb joint angles. However, as most of these studies do not report absolute reliability statistics, comparisons cannot be established. Only the SEMs of the UG and the digital inclinometer for knee flexion have been reported [23], and these were approximately four times higher than the VS SEMs.

The inter-rater reliability of the VS also revealed excellent ICCs (>0.88) and low SEMs and MDCs. Once again, the differences between movements were minimal (SEM% < 4.35%), and only one test showed an ICC below 0.9. Nevertheless, the ICCs were lower for hip adduction than hip abduction, and also for ankle dorsiflexion when compared to plantarflexion. These differences between the two movements of the same plane are consistent with previous studies that found higher inter-rater reliability for knee flexion compared with extension [21]. Again, this could be explained by the large differences between their angles.

Our study showed that the intra-rater reliability of the VS was similar to the inter-rater reliability for almost all movements. This finding does not correlate with previous reliability studies, which, overall, found that the intra-rater reliability of the goniometer is slightly higher than its inter-rater reliability [20,21]. In our study, only ankle dorsiflexion and hip adduction movements showed slightly lower inter-rater reliability (i.e., lower ICCs and higher SEM%) than intra-rater reliability. In our opinion, these slight differences are not necessarily due to the examiner’s experience with the VS. In fact, our study showed that an inexperienced examiner receiving basic training with the VS can achieve high inter-rater reliability for all movements.

Despite its novel findings, this study was subject to limitations. The main limitation was that the measurements were made on healthy participants; therefore, the results cannot be generalized in clinical contexts. Thus, future research should evaluate the reliability of this method on other populations.

## 5. Conclusions

Assessments of ROM in the lower limb joints are an essential aspect of clinical examinations. To this end, different tools are used, from the goniometer as a reference tool to other novel methods. The VS has been developed to track trajectories via a camera and for evaluating ROM. Our study demonstrates that the VS is a valid and reliable measurement tool that can be easily used in healthy adults.

## Figures and Tables

**Table 1:** Characteristics of the participants (*n* = 35).

	Mean (SD)	Range
Age (years)	34.8 (12.5)	18–61
Body mass (kg)	74.9 (20.7)	54–170
Stature (cm)	171.4 (8.6)	156–188
BMI (kg/m^2^)	25.3 (5.9)	20.6–56.1
Gender	Males (*n* = 19)	Females (*n* = 16)
Laterality	Right (*n* = 30)	Left (*n* = 5)

**Table 2:** Validity between Veloflex and goniometer to measure the range of motion of the lower limb joints.

Movement	Goniometer (SD)	VeloFlex (SD)	Pearson Coefficient	Lo A− (%)	Lo A+ (%)	Mean Difference (%)	SD Difference (%)
HF	111.70° (7.34°)	111.81° (7.34°)	0.999	−0.62° (−0.6%)	0.83° (0.7%)	0.11° (0.1%)	0.37° (0.3%)
HE	23.96° (4.47°)	23.92° (4.53°)	0.998	−0.67° (−2.7%)	0.60° (2.4%)	−0.04° (−0.2%)	0.32° (1.4%)
HIR	42.04° (5.06°)	42.09° (5.06°)	0.998	−0.57° (−1.3%)	0.69° (1.6%)	0.06° (0.1%)	0.32° (0.8%)
HER	39.44° (3.91°)	39.56° (3.91°)	0.995	−0.64° (−1.6%	0.89° (2.2%)	0.12° (0.3%)	0.39° (1%)
HAD	22.41° (3.05°)	22.63° (2.92°)	0.995	−0.39° (−1.7%)	0.83° (3.7%)	0.22° (1.1%)	0.31° (1.5%)
HAB	44.21° (5.24°)	44.01° (5.26°)	0.997	−0.96° (−2.1%)	0.56° (1.2%)	−0.20° (−0.5%)	0.39° (0.9%)
KF	139.88° (7.75°)	139.97° (7.84°)	0.999	−0.66° (−0.4%)	0.85° (0.6%)	0.09° (0.06%)	0.39° (0.3%)
ADF	20.27° (2.83°)	20.42° (2.84°)	0.992	−0.55° (−2.6%)	0.85° (4.1%)	0.15° (0.8%)	0.36° (1.8%)
APF	42.28° (4.43°)	42.36° (4.26°)	0.995	−0.79° (−1.8%)	0.96° (2.2%)	0.09° (0.3%)	0.45° (1.2%)

SD: Standard deviation; LoA: Limit of agreement; HF: Hip flexion; HE: Hip extension; HIR: Hip internal rotation; HER: Hip external rotation; HAD: Hip adduction; HAB: Hip abduction; KF: Knee flexion; ADF: Ankle dorsiflexion; APF: Ankle plantarflexion.

**Table 3:** Intra-rater reliability of Veloflex to measure the range of motion of the lower limb joints.

Movement	Retest (SD)	ICC (95% CI)	SEM (SEM%)	MDC
HF	111.37° (−7.81°)	0.97 (0.95 to 0.99)	1.17° (1.05%)	3.26°
HE	23.40° (−4.43°)	0.98 (0.95 to 0.99)	0.24° (1.03%)	0.68°
HIR	41.30° (−5.13°)	0.98 (0.96 to 0.99)	0.78° (1.90%)	2.17°
HER	39.48° (−4.55°)	0.96 (0.93 to 0.98)	0.79° (1.99%)	2.18°
HAD	22.26° (−3.21°)	0.96 (0.93 to 0.98)	0.59° (2.61%)	1.63°
HAB	44.30° (−5.65°)	0.99 (0.97 to 0.99)	0.87° (1.97%)	2.42°
KF	139.43° (−7.85°)	0.99 (0.98 to 0.99)	0.74° (0.53%)	2.06°
ADF	20.06° (−2.70°)	0.93 (0.87 to 0.97)	0.70° (1.71%)	1.95°
APF	41.85° (−4.39°)	0.98 (0.95 to 0.99)	0.65° (1.54%)	1.81°

SD: Standard deviation; ICC: Intraclass correlation coefficient; CI: Confidence interval; SEM: Standard error of measurement; MDC: Minimum detectable change; HF: Hip flexion; HE: Hip extension; HIR: Hip internal rotation; HER: Hip external rotation; HAD: Hip adduction; HAB: Hip abduction; KF: Knee flexion; ADF: Ankle dorsiflexion; APF: Ankle plantarflexion.

**Table 4:** Inter-rater reliability of Veloflex to measure the range of motion of the lower limb joints.

Movement	Examiner 2 (SD)	ICC (95% CI)	SEM (SEM%)	MDC
HF	111.31° (−7.03°)	0.95 (0.91 to 0.97)	1.60° (1.43%)	4.42°
HE	23.64° (−4.39°)	0.95 (0.91 to 0.98)	0.93° (3.95%)	2.57°
HIR	41.90° (−5.42°)	0.96 (0.91 to 0.98)	1.10° (2.64%)	3.04°
HER	39.29° (−4.10°)	0.94 (0.88 to 0.97)	1.07° (2.72%)	2.96°
HAD	22.40° (3.24°)	0.91 (0.82 to 0.95)	0.97° (4.35%)	2.69°
HAB	42.78° (−5.14°)	0.98 (0.95 to 0.99)	0.82° (1.88%)	2.27°
KF	138.73° (−7.93°)	0.98 (0.96 to 0.99)	1.13° (0.81%)	3.13°
ADF	20.17° (−2.79°)	0.88 (0.76 to 0.94)	0.96° (4.76%)	2.65°
APF	41.71° (−4.17°)	0.95 (0.91 to 0.98)	0.91° (2.18%)	2.52°

SD: Standard deviation; ICC: Intraclass correlation coefficient; CI: Confidence interval; SEM: Standard error of measurement; MDC: Minimum detectable change; HF: Hip flexion; HE: Hip extension; HIR: Hip internal rotation; HER: Hip external rotation; HAD: Hip adduction; HAB: Hip abduction; KF: Knee flexion; ADF: Ankle dorsiflexion; APF: Ankle plantarflexion.
